# Bioinformatics and system biology approach to identify potential common pathogenesis for COVID-19 infection and osteoarthritis

**DOI:** 10.1038/s41598-023-32555-y

**Published:** 2023-06-08

**Authors:** Ziyi Chen, Wenjuan Wang, Hao Jue, Yinghui Hua

**Affiliations:** Department of Sports Medicine, Huashan Hospital, Fudan University, No. 12, Wulumuqi Zhong Road, Shanghai, 200040 People’s Republic of China

**Keywords:** Computational biology and bioinformatics, Biomarkers, Diseases, Pathogenesis, Risk factors

## Abstract

A growing of evidence has showed that patients with osteoarthritis (OA) had a higher coronavirus 2019 (COVID-19) infection rate and a poorer prognosis after infected it. Additionally, scientists have also discovered that COVID-19 infection might cause pathological changes in the musculoskeletal system. However, its mechanism is still not fully elucidated. This study aims to further explore the sharing pathogenesis of patients with both OA and COVID-19 infection and find candidate drugs. Gene expression profiles of OA (GSE51588) and COVID-19 (GSE147507) were obtained from the Gene Expression Omnibus (GEO) database. The common differentially expressed genes (DEGs) for both OA and COVID-19 were identified and several hub genes were extracted from them. Then gene and pathway enrichment analysis of the DEGs were performed; protein–protein interaction (PPI) network, transcription factor (TF)-gene regulatory network, TF-miRNA regulatory network and gene-disease association network were constructed based on the DEGs and hub genes. Finally, we predicted several candidate molecular drugs related to hub genes using DSigDB database. The receiver operating characteristic curve (ROC) was applied to evaluate the accuracy of hub genes in the diagnosis of both OA and COVID-19. In total, 83 overlapping DEGs were identified and selected for subsequent analyses. *CXCR4*, *EGR2*, *ENO1*, *FASN*, *GATA6*, *HIST1H3H*, *HIST1H4H*, *HIST1H4I*, *HIST1H4K*, *MTHFD2*, *PDK1*, *TUBA4A*, *TUBB1* and *TUBB3* were screened out as hub genes, and some showed preferable values as diagnostic markers for both OA and COVID-19. Several candidate molecular drugs, which are related to the hug genes, were identified. These sharing pathways and hub genes may provide new ideas for further mechanistic studies and guide more individual-based effective treatments for OA patients with COVID-19 infection.

## Introduction

Osteoarthritis (OA) is a degenerative joint disease, exerting a substantial health burden on individuals, society and governments^1,2^. With women and old people disproportionately affected, OA affects more than 500 million people worldwide^1,3^. The etiology of OA is complicated and is currently believed to be related to a combination of factors such as biomechanical processes, trauma, chronic inflammation, and immune response, etc., which still needs to be explored^1,4–7^.

Coronavirus disease 2019 (COVID-19), caused by severe acute respiratory syndrome coronavirus 2 (SARS-CoV-2), emerged at the end of 2019 and has been ravaging the world from then on^8,9^. There have been 760,360,956 confirmed cases and 6,873,477 deaths of COVID-19 globally up to 16 March 2023 (https://covid19.who.int/).

COVID-19 has placed a severe health burden on individuals, especially those with underlying medical conditions, such as OA^9,10^. According to a cross-sectional study in Barcelona, OA patients had a higher percentage of COVID-19 infection and a poorer prognosis if infecting COVID-19 in the long term comparing to the general health population^11^. Furthermore, Fredi et al. reported that seniority and comorbidities were associated with poorer prognosis of COVID-19 infection in patients with rheumatic and musculoskeletal diseases^12^. In another prospective cohort study, COVID-19 outcomes were worse in inflammatory arthritis patients treated with glucocorticoids^13^. Moreover, it has been reported that COVID-19 patients would develop some symptoms such as joint pain, muscle discomfort and bone demineralization which resembled clinical manifestations of early OA^14^.

Some possible mechanism might account for the epidemiological phenomenon. As is known to all, OA often coexists with other chronic diseases such as obesity, cardiovascular disease and diabetes which are all risk factors for COVID-19 infection. For example, in OA individuals with obesity, the pre-existing inflammatory state could accelerate or potentiate SARS-CoV-2 infection via the NLRP3 inflammasome activation and the release of pro-inflammatory cytokines^15^. Additionally, patients suffering from COVID-19 often presented with hypocalcemia, vitamin D deficiency, and immobility due to the diseases all contributing to bone demineralization which is the typical symptoms of early OA^14,16^. Scientists also found that the systematic inflammation, the overstimulation of the immune response contributing to endothelial and adipose tissue dysfunction, and neuronal sensitization could be the trigger for OA-like changes in COVID-19 patients^14^. Additionally, a common neutrophil activation characteristics, renin-angiotensin system (RAS) perturbation and the onset of “cytokine storm” shared by severe COVID-19 and other acute inflammatory states might also be the underlined mechanism which need to be clarified in the future^17,18^.

Investigating the common gene profiles of both OA and COVID-19 patients may shed light on the underlying common pathogenesis of these two diseases. Our study was the first research aiming to identify key genes associated with the pathogenesis of OA complicated with COVID-19. Integrated bioinformatics and system biology analysis were used to determine common differentially expressed genes (DEGs) and identify hub genes of both COVID-19 and OA. Function annotation, PPI network, transcription factor (TF)-gene regulatory network, microRNA (miRNA)-gene regulatory network and gene-disease association network were constructed based on the DEGs and hub genes. These hub genes may provide new ideas for further biological mechanistic studies and help discover novel therapeutic targets for OA patients with COVID-19 infection.

## Materials and methods

### Datasets preparation

GSE147507 and GSE51588 datasets were downloaded from the Gene Expression Omnibus (GEO, http://www.ncbi.nlm.nih.gov/geo/) database. GSE147507 dataset includes 23 COVID-19 lung biopsy samples and 55 non-COVID-19 controls applying high throughput sequencing Illumina NextSeq 500 platform for extracting RNA sequence^19^. GSE51588 dataset contains 40 OA subchondral bone samples and 10 healthy controls which used Agilent microarray platform^20^.

### Identification of DEGs and shared DEGs between COVID-19 and OA

We used “limma” package of R software (version 4.1.1) to select DEGs between COVID-19 and non-COVID-19, and between OA and normal^21^. The criteria for screening out DEGs were set as P < 0.05 and |log fold change (FC)|> 1^21,22^. “Pheatmap”, “EnhancedVolcano” and “ggplot2” packages of R software (version 4.1.1) were applied to generate heatmaps and volcano plots^23–25^. Then the common DEGs of GSE147507 and GSE51588 were acquired using jvenn, an online VENN analysis tool (http://jvenn.toulouse.inra.fr/app/example.html)^26^.

### Gene ontology (GO) and pathway enrichment analysis

GO (biological processes, cellular component, and molecular functions) and pathway enrichment analysis (WikiPathways, Reactome, BioCarta, and Kyoto Encyclopedia of Genes and Genomes (KEGG)) were conducted using EnrichR online tool (https://maayanlab.cloud/Enrichr/) to specify the shared function and pathways between COVID-19 and OA^27^. The P value < 0.05 was considered significantly enriched.

### PPI network analysis

STRING (http://string-db.org) (version 11.5) is a database for the study of protein–protein association networks with increased information coverage on more than 14,000 species, 67 million proteins and 20 billion interactions, supporting functional discovery in genome-wide experimental datasets^28^. We constructed the PPI network of proteins derived from shared DEGs utilizing the STRING repository with an interaction score > 0.15.

### Identification and analysis of hub genes

In the PPI network which consists of nodes, edges and their connections, nodes with the most entanglement are considered as hub genes. Cytohubba (http://apps.cytoscape.org/apps/cytohubba) is a novel plugin of Cytoscape for extracting central elements of a biological network^29^. The hub genes were selected by applying seven algorithms (Closeness, MCC, Degree, MNC, Radiality, Stress and EPC) and intersecting them.

GeneMANIA (https://genemania.org)^30^, a flexible user-friendly web site for analyzing gene function, was utilized to construct a co-expression network of identified hub genes.

### Construction of TF-gene and miRNA-gene regulatory network

TFs govern gene transcription information and miRNAs post-transcriptionally control gene expression; hence, their activity is essential for gaining molecular insights^31,32^. NetworkAnalyst is a broad online platform for statistics, visualization, and meta-analysis of web-based gene expression data^33^. JASPAR (http://jaspar.genereg.net) is a public resource, provides across six classification group of TF combining spectrum of multiple species^34^. MirTarbase is a tool which helps researchers filter top miRNAs and detect biological functions and features that facilitate the development of biological hypotheses^35^. We topologically located credible TFs which tend to bind to our hub genes from the JASPAR database on the networkAnalyst platform. Then we extracted miRNAs that interacted with our hub genes from mirTarbase via networkAnalyst.

### Gene-disease association analysis

DisGeNET is a comprehensive platform that integrates information on genes and variants associated with human diseases and can be used to investigate the molecular basis of specific human diseases and their comorbidities^36^. We also examined the gene-disease relationship using DisGeNET database via NetworkAnalyst to disclose associated diseases and their complications with hub genes.

### Evaluation of applicant drugs

Drug Signatures database (DSigDB) containing 22,527 gene sets was used to generate the small molecules which could downregulate the expression of hub genes^37^. The access to the DSigDB database is acquired through Enrichr (https://amp.pharm.mssm.edu/Enrichr/) platform^38^. Drug molecules were identified using the DSigDB via Enrichr based on the selected hub genes.

### ROC curves of hub genes

The receiver operating characteristic (ROC) curve was drawn and the area under the curve (AUC) of the ROC curve was calculated using the pROC package in R in order to detect the diagnostic ability of all candidate hub genes, respectively^39^.

## Results

### Identification of DEGs of COVID-19 and OA

**Figure 1:**
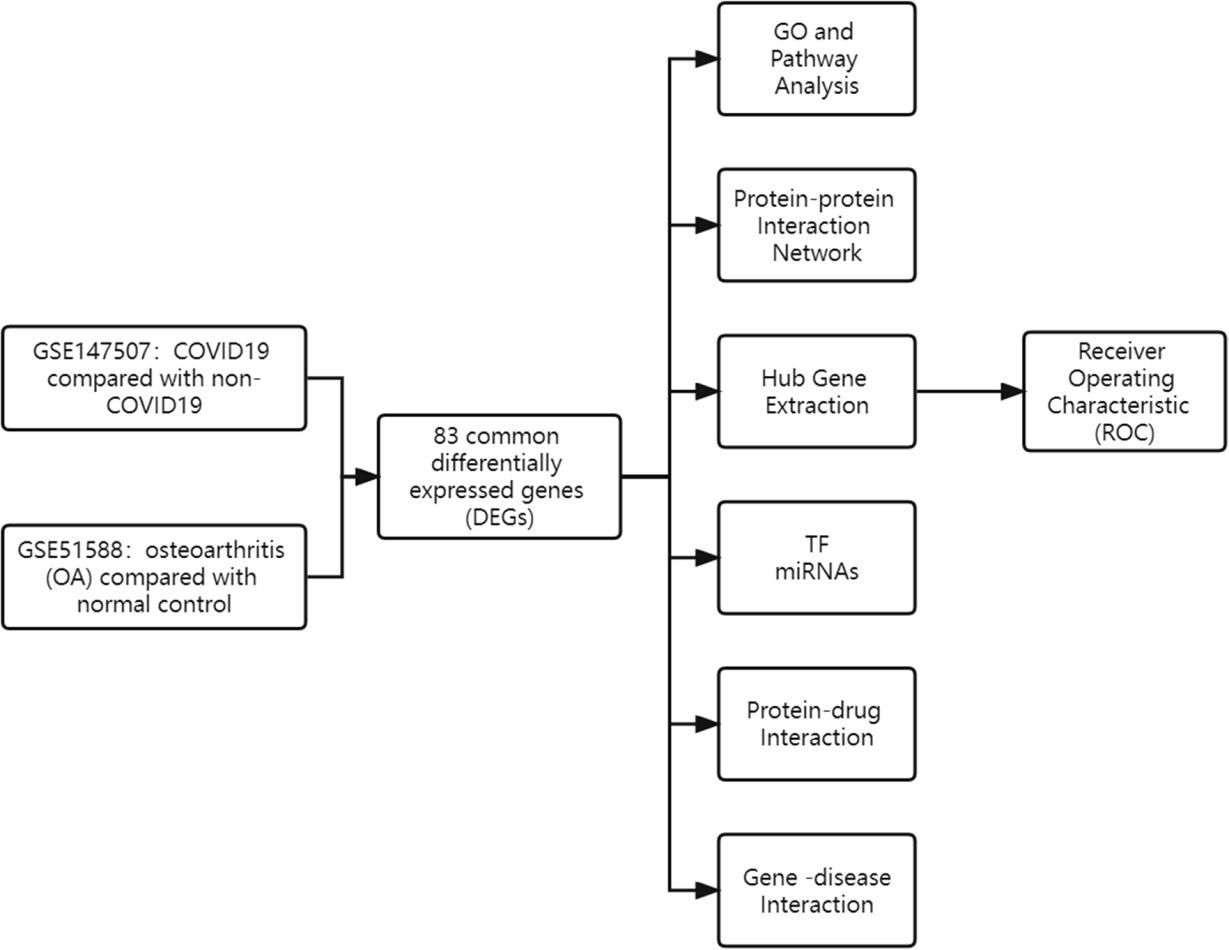
Schematic illustration of the overall general workflow of this study.

**Figure 2:**
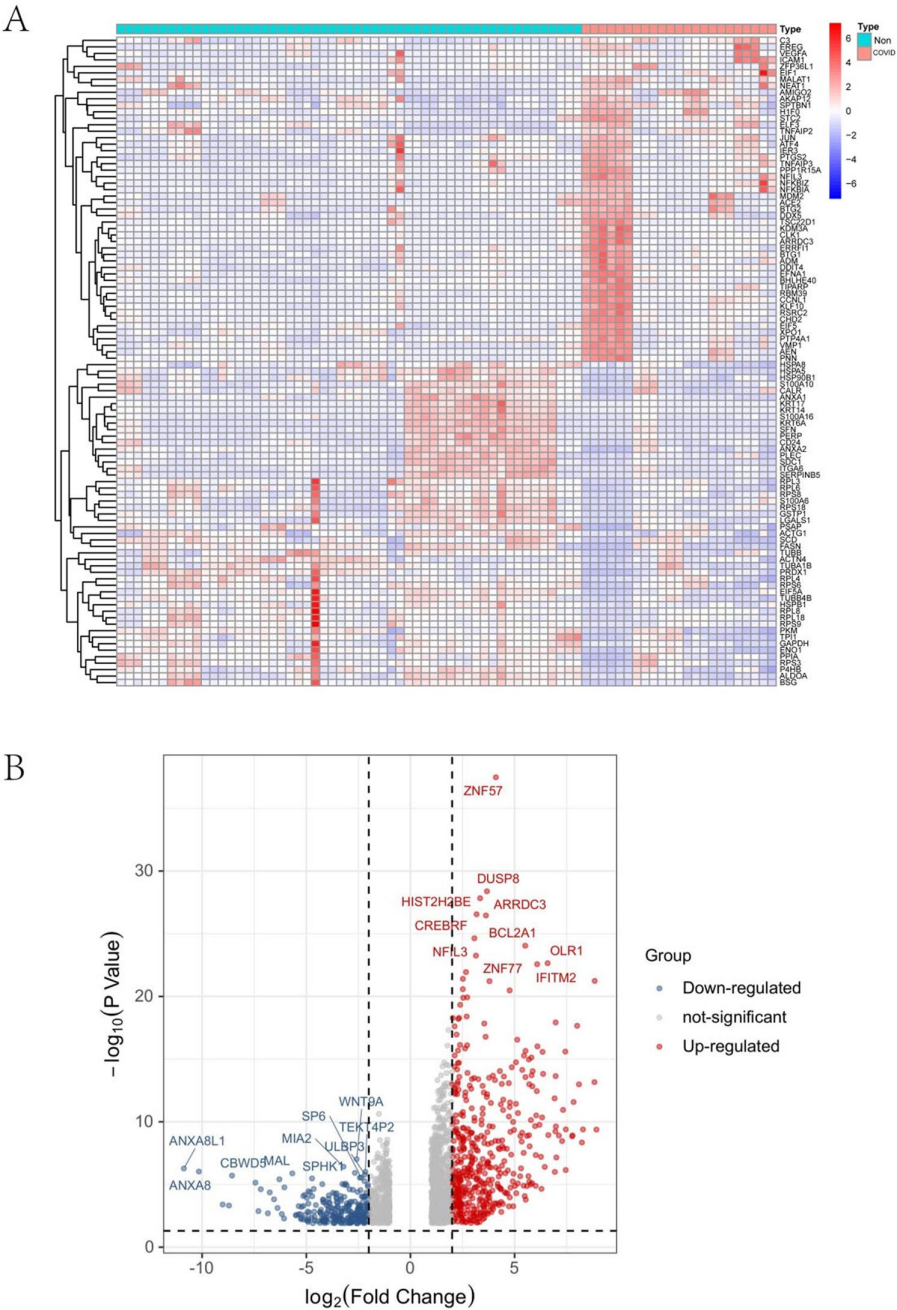
Expression characteristics of DEGs in COVID-19 patient. (**A**) Heat map and (**B**) Volcano plot present the identified DEGs between COVID-19 patients and normal controls (|logFC|> 1 and P-value < 0.05 were defined as screening standard to obtain DEGs for COVID-19). Blue represents low expression values, and red represents high expression values. *DEGs* differentially expressed genes, *COVID-19* Coronavirus 2019.

**Figure 3:**
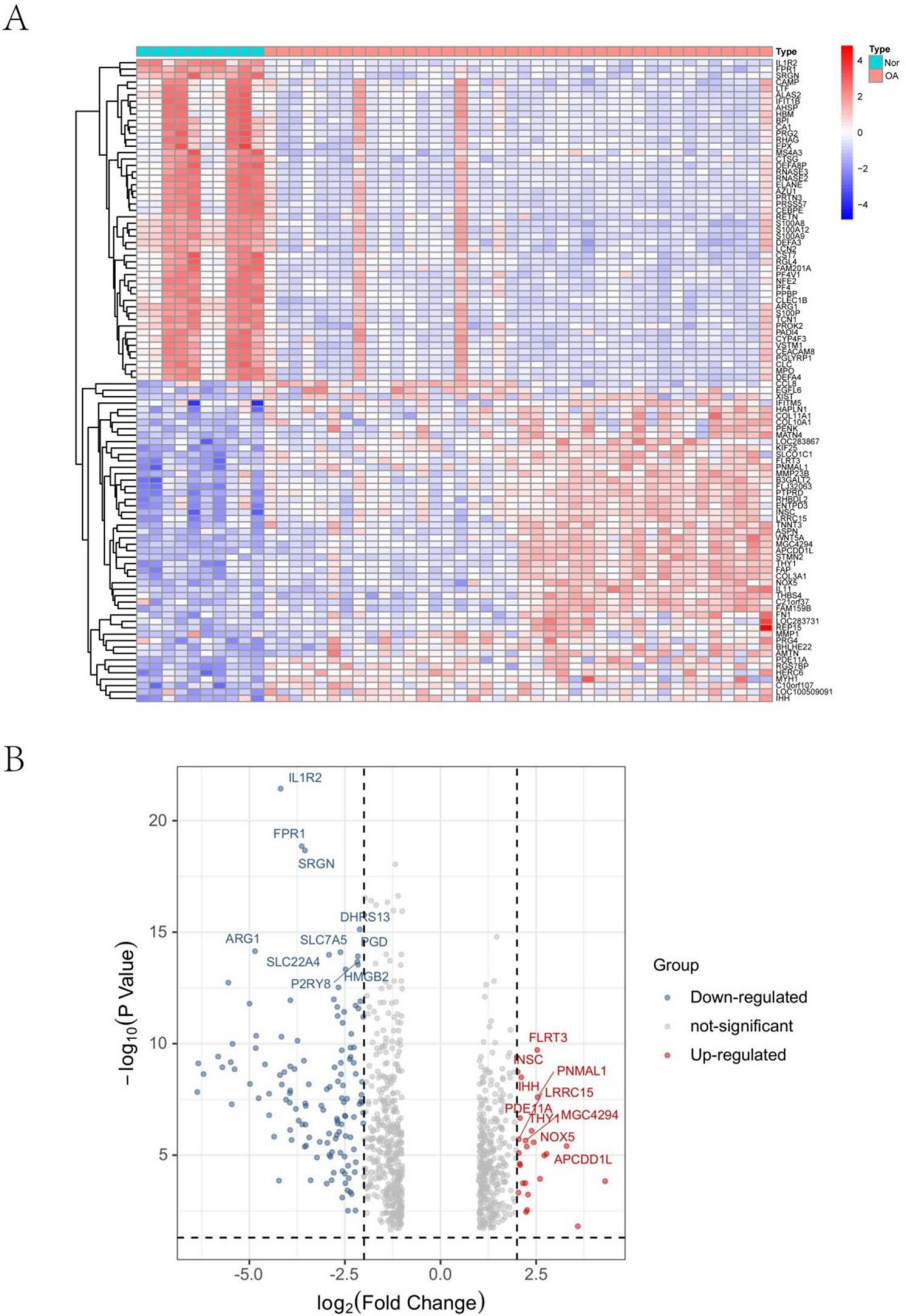
Expression characteristics of DEGs in OA. (**A**) Heat map and (**B**) Volcano plot present the identified DEGs between OA patients and normal controls (|logFC|> 1 and P-value < 0.05 were defined as screening standard to obtain DEGs for OA). Blue represents low expression values, and red represents high expression values. *DEGs* differentially expressed genes, *OA* osteoarthritis.

**Figure 4:**
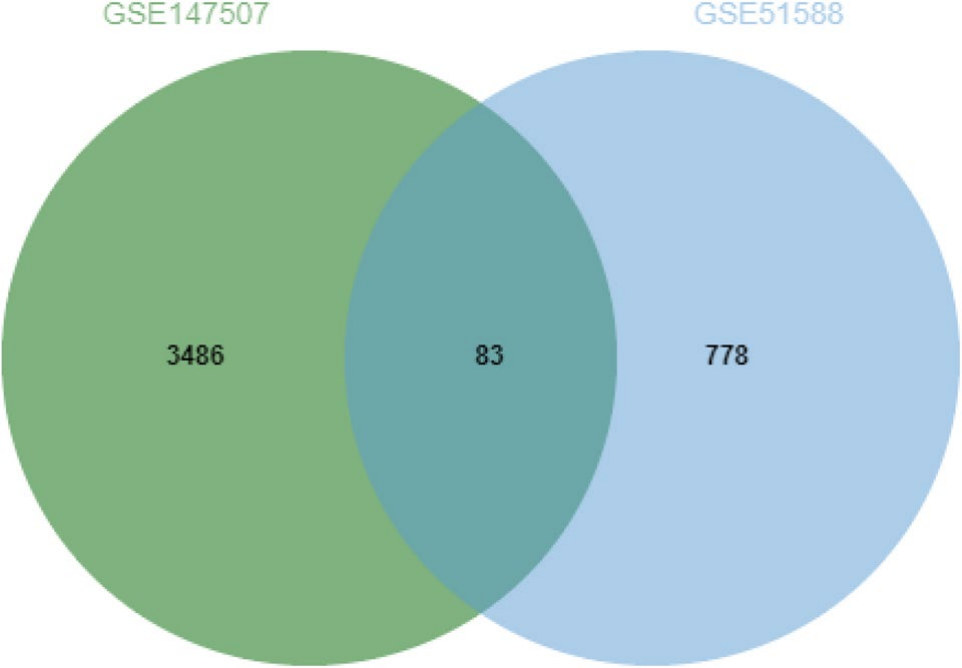
Venn diagram showing the two datasets owning an overlap of 83 DEGs. *DEGs* differentially expressed genes.

The overall flow chart of this study was shown in Fig. 1. 3569 DEGs were identified in the GSE147507 dataset, and 861 DEGs were figured out in the GSE51588 dataset. Figures 2 and 3 displayed the distribution of DEGs between COVID-19 and non-COVID-19 patients, and between OA patients and normal controls through a heatmap and a volcano plot, respectively. The intersection of DEGs of GSE147507 and GSE51588 datasets was visualized by Venn diagrams, and there were 83 shared DEGs selected (Fig. 4).

### Gene ontology and pathway enrichment analysis

**Figure 5:**
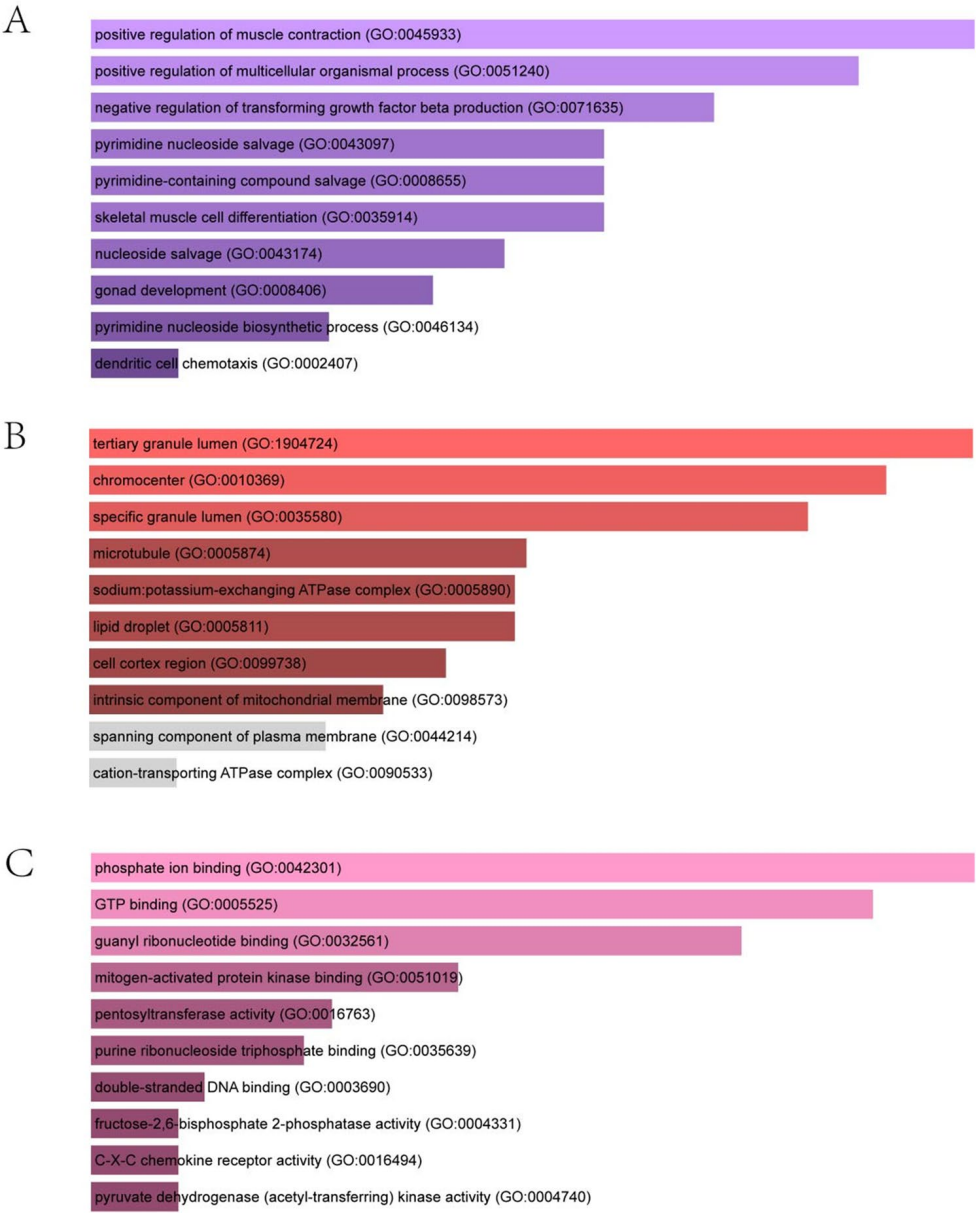
The bar graphs of ontological analysis of shared DEGs between COVID-19, and OA: (**A**) biological processes, (**B**) molecular function, and (**C**) cellular component. *DEGs* differentially expressed genes, *COVID-19* Coronavirus 2019, *OA* osteoarthritis.

**Figure 6:**
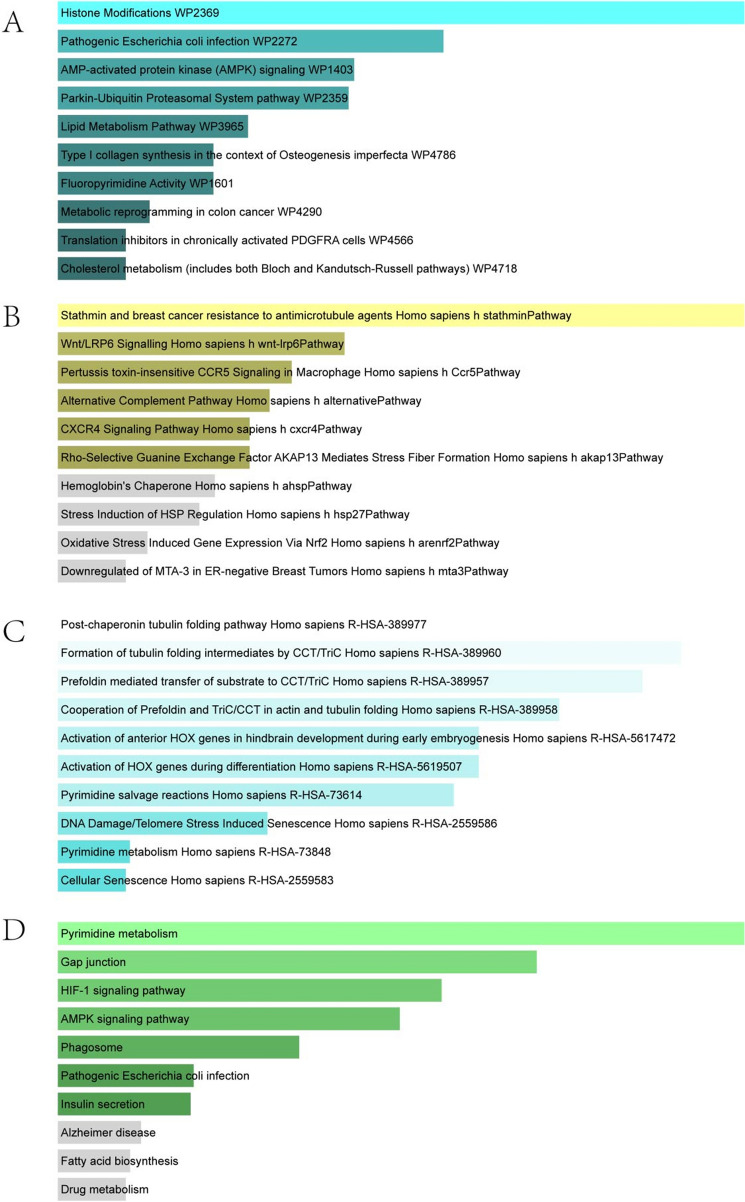
The bar graphs of pathway enrichment analysis of shared DEGs between COVID-19, and OA: (**A**) wikipathway, (**B**) biocarta pathway, (**C**) reactome pathway, and (**D**) KEGG 2021 human pathway^65^. *DEGs* differentially expressed genes, *COVID-19* Coronavirus 2019, *OA* osteoarthritis.

The most significant terms in the biological process, molecular functions, and cellular components category of GO were positive regulation of muscle contraction, tertiary granule lumen and phosphate iron binding, respectively (Fig. 5). The enriched pathways of the common DEGs between COVID-19 and OA were gathered from four global databases, including KEGG, WikiPathways, Reactome, and BioCarta, and visualized in Fig. 6.

### PPI network analysis

**Figure 7:**
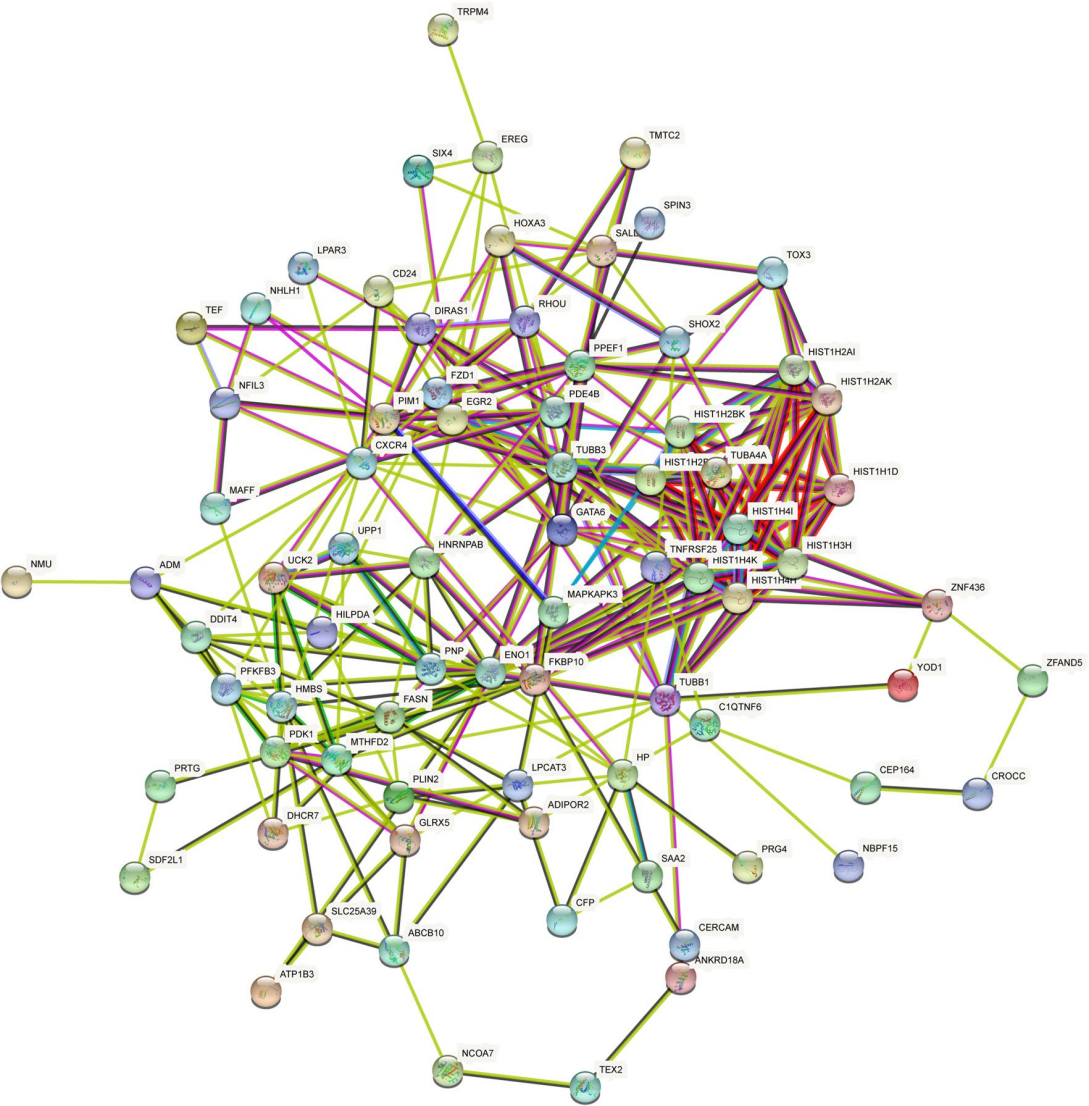
PPI network of common DEGs between COVID-19 and OA. *PPI* protein–protein interwork, *DEGs* differentially expressed genes, *COVID-19* Coronavirus 2019, *OA* osteoarthritis.

We carefully studied and visualized the PPI network in STRING to predict the interaction and adhesion pathways of common DEGs. The PPI network of common DEGs consisted of 81 nodes and 273 edges, where the PPI enrichment P value was lower than 1.75E−08, as shown in Fig. 7.

### Classification and function analysis of hub genes

**Figure 8:**
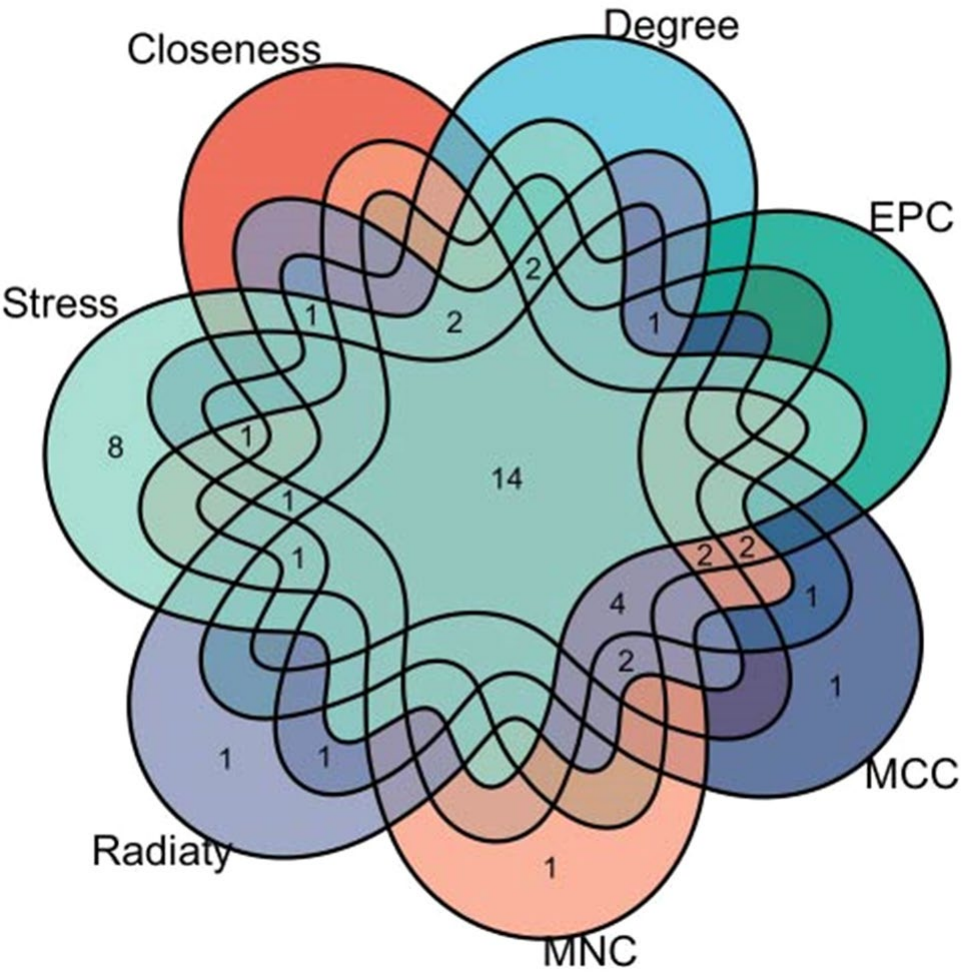
The Venn diagram showed 12 overlapping hub genes screened by 7 algorithms.

Seven algorithms of plugin cytoHubba in Cytoscape were applied to screen out the top 30 hub genes. 14 common hub genes were finally identified through the intersection of Venn diagrams, including *CXCR4*, *EGR2*, *ENO1*, *FASN*, *GATA6*, *HIST1H3H*, *HIST1H4H*, *HIST1H4I*, *HIST1H4K*, *MTHFD2*, *PDK1*, *TUBA4A*, *TUBB1* and *TUBB3* (Fig. 8).

**Figure 9:**
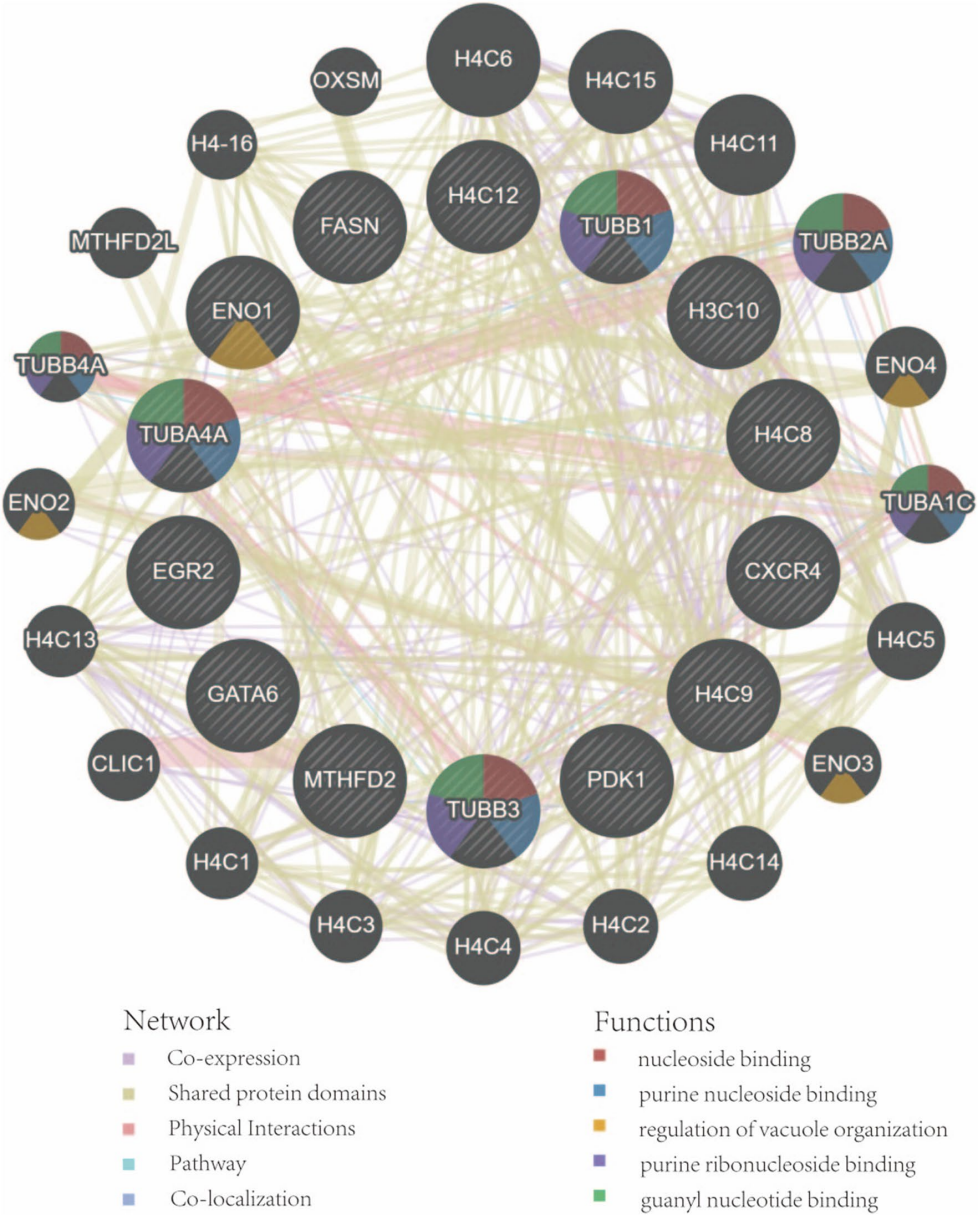
Hub genes and their co-expression genes were analyzed via GeneMANIA.

Based on GeneMANIA database, we constructed a complex gene interaction network to decipher the biological functions of these hub genes (Fig. 9), with the co-expression of 63.34%, shared protein domains (17.75%), physical interactions of 17.58%, pathway of 0.75% and co-localization of 0.57%. The results also demonstrated that they were mainly related to the nucleoside binding, purine nucleoside binding, regulation of vacuole organization, purine ribonucleoside binding, guanyl nucleotide binding. These hub genes can be potential biomarkers, which may also lead to new therapeutic strategies for investigated diseases.

### Determination of regulatory signatures

**Figure 10:**
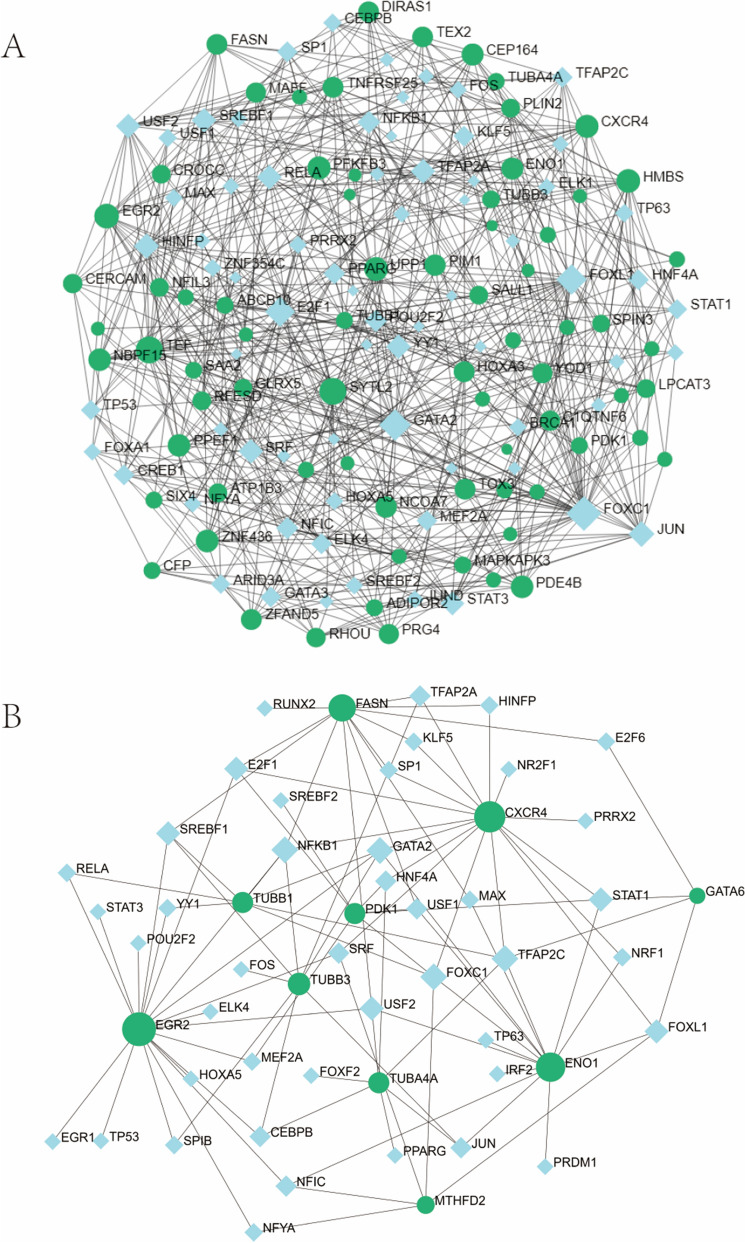
The DEG-TF (**A**) and hub gene-TF (**B**) regulatory interaction network. Herein, the square nodes are TFs, and gene symbols interact with TFs as circle nodes. *DEGs* differentially expressed genes, *TF* transcription factors.

**Figure 11:**
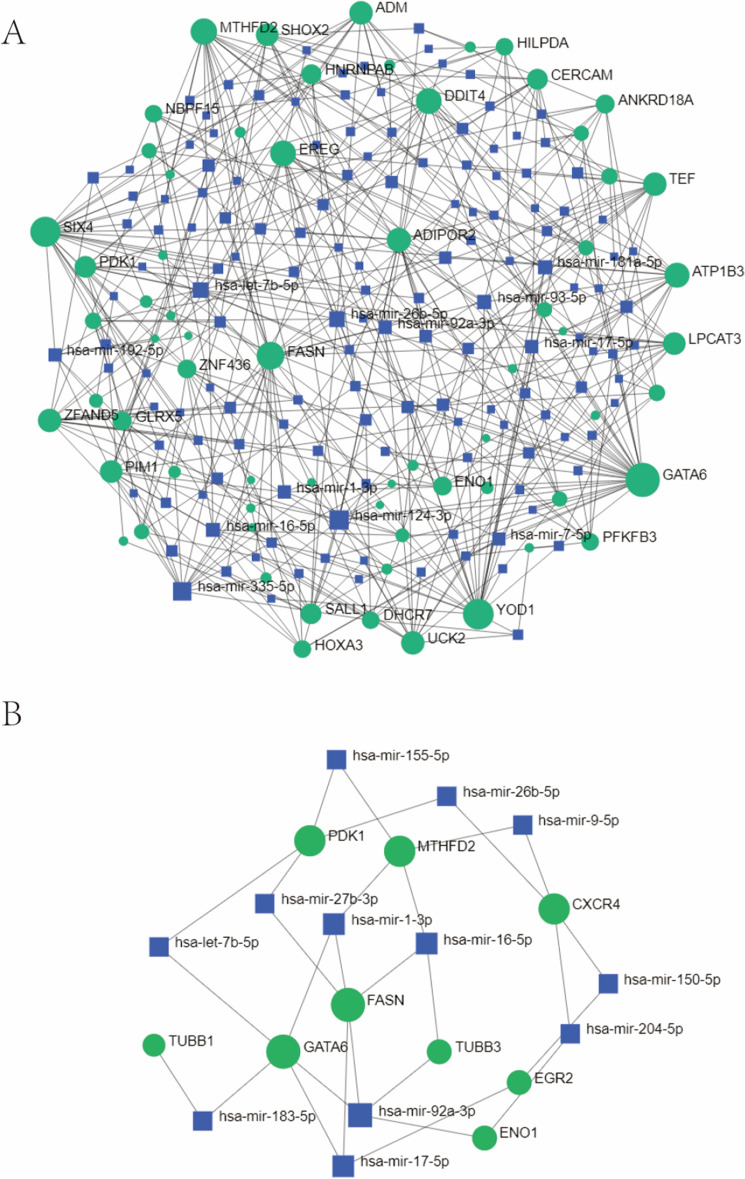
The DEG–miRNA (**A**) and hub gene-miRNA (**B**) regulatory interaction network. Herein, the square node indicates miRNAs and gene symbols interact with miRNAs as a circle shape. *DEGs* differentially expressed genes, *miRNAs* microRNAs.

TFs and miRNAs interacting with both the DEGs and 14 hub genes were predicted and visualized in Networkanalyst, respectively (Figs. 10, 11). From TFs–genes and miRNA–gene interaction network analysis, it has been ascertained that 71 TFs and 145 miRNAs regulatory signatures regulate with more than one common DEGs. The TF–gene interaction network of 14 hub genes included 53 nodes, 99 edges and 10 genes, while the miRNA–gene interaction network contained 21 nodes, 29 edges and 9 genes.

### Identification of disease association

**Figure 12:**
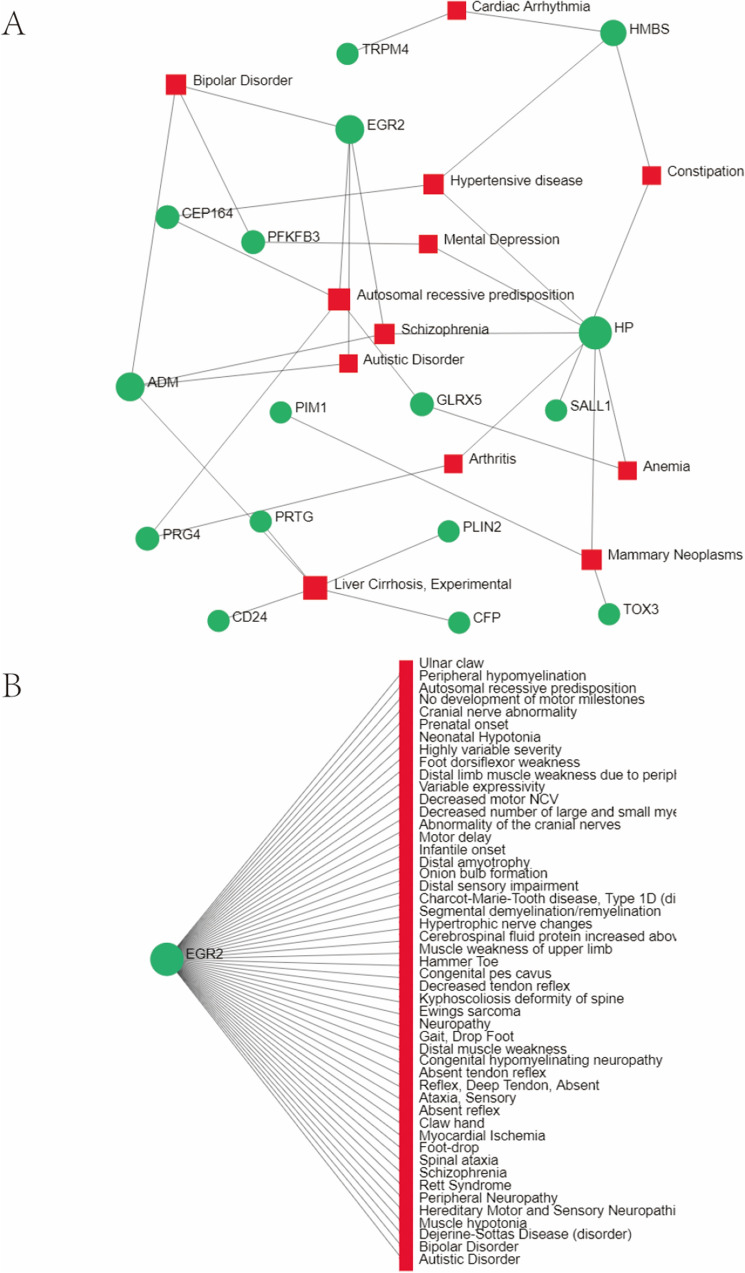
The gene-disease association network represents diseases associated with mutual DEGs and hub genes. The disorder depicted by the square node and also its subsequent gene symbols is defined by the circle node. *DEGs* differentially expressed genes.

Treatment design strategies for disease open the door to reveal the relationship between genes and diseases. Through the analysis of the gene-disease association by Networkanalyst, we found that arthritis, mammary neoplasms, liver cirrhosis, anemia, autistic disorder, schizophrenia, autosomal recessive predisposition, mental depression, hypertensive disease, bipolar disorder, constipation and cardiac arrhythmia are most related to our hub genes. The association between gene-disease is displayed in Fig. 12.

### Identification of candidate drugs

**Figure 13:**
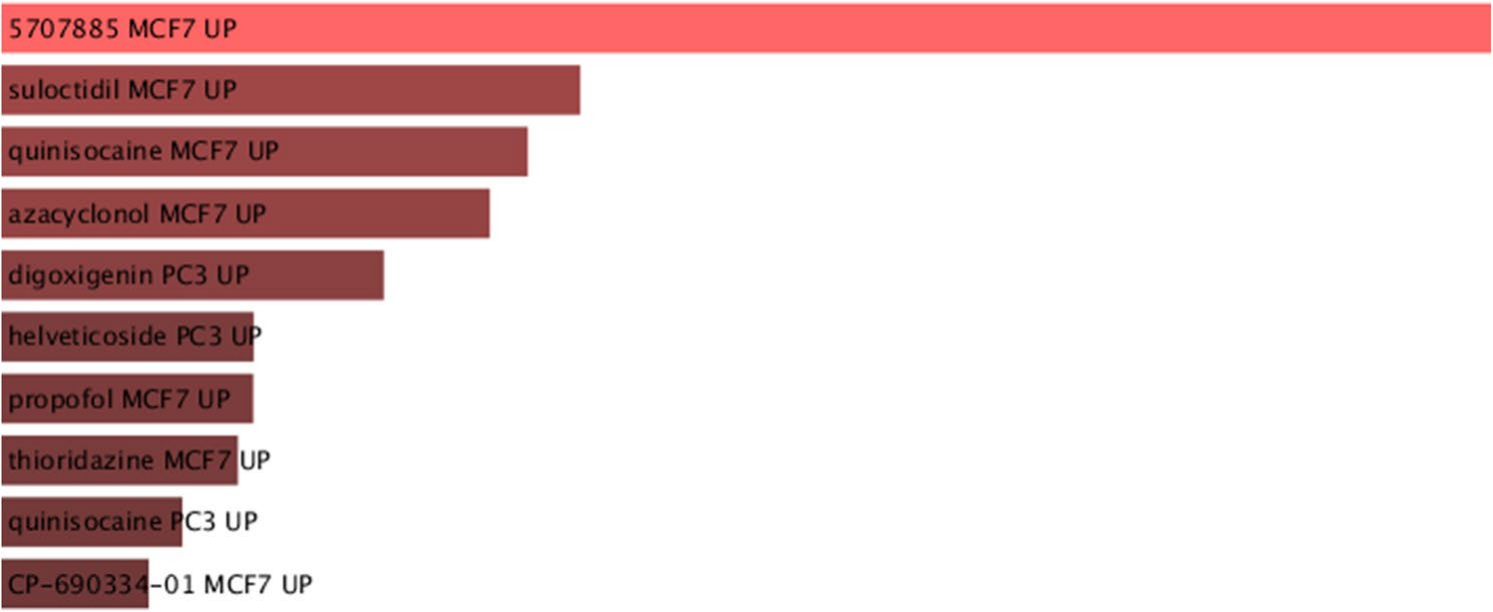
List of the top 10 suggested drugs for patients with both COVID-19 and OA. *COVID-19* Coronavirus 2019, *OA* osteoarthritis.

Small molecule drugs regulating the expression of hub genes were collected from the DSigDB database on Enrichr platform. The results from the potential small molecules were generated based on their P values to represent the closeness between the small molecules and genes. Table 1 and Fig. 13 pointed out the top 10 potential small molecule drugs for hub genes.

**Table 1:** List of the top 10 suggested drugs for patients with both COVID-19 and OA.

Term	Overlap	P-value	Adjusted P-value	Odds ratio	Genes
Strophanthidin PC3 *UP*	17/459	5.132672800037316E−12	6.759730077649145E−9	11.349067599067599	PFKFB3;HIST1H2AI;HIST1H4K;HIST1H2AK;ZFAND5;HIST1H2BK;GATA6;CXCR4;ADM;PNP;NFIL3;DDIT4;MAFF;HIST1H4H;HIST1H3H;HIST1H4I;HIST1H2BD
Dimethyloxalylglycine PC3 *UP*	8/48	2.053283101851948E−11	1.3266193980150453E−8	53.00533333333333	PFKFB3;HILPDA;MAFF;DDIT4;CXCR4;ADM;PLIN2;PDK1
Cephaeline PC3 *UP*	17/514	3.0484121070337935E−11	1.3266193980150453E−8	10.064630205475275	EGR2;HIST1H2AI;HIST1H4K;HIST1H2AK;GATA6;ADM;NFIL3;DDIT4;MAFF;HIST1H1D;PIM1;PDE4B;HIST1H4H;HIST1H3H;HIST1H4I;UPP1;HIST1H2BD
Cicloheximide PC3 *UP*	15/378	4.029216091161869E−11	1.3266193980150453E−8	11.882596013612057	EGR2;HIST1H2AI;HIST1H4K;HIST1H2AK;HIST1H2BK;GATA6;ADM;NFIL3;MAFF;PIM1;PDE4B;HIST1H4H;HIST1H3H;HIST1H4I;HIST1H2BD
helveticoside PC3 *UP*	14/324	6.267362844743696E−11	1.4159213027729708E−8	12.833006077606358	EGR2;HIST1H2AI;HIST1H4K;HIST1H2AK;GATA6;ADM;YOD1;MAFF;HIST1H1D;PIM1;HIST1H4H;HIST1H3H;HIST1H4I;HIST1H2BD
Anisomycin PC3 *UP*	21/899	7.035776704585525E−11	1.4159213027729708E−8	7.34475347196708	EGR2;PFKFB3;HIST1H2AI;HIST1H4K;HIST1H2AK;HIST1H2BK;GATA6;CXCR4;ADM;YOD1;PNP;NFIL3;MAFF;HIST1H1D;PIM1;PDE4B;HIST1H4H;HIST1H3H;HIST1H4I;UPP1;HIST1H2BD
5,707,885 MCF7 *UP*	8/56	7.525777615346086E−11	1.4159213027729708E−8	44.153333333333336	NFIL3;HIST1H4K;DDIT4;MAFF;HIST1H4H;HIST1H3H;HIST1H4I;ENO1
Azacyclonol MCF7 *UP*	10/126	1.1309520631813494E−10	1.8618298340122964E−8	23.383325460557394	EGR2;HIST1H2AI;NFIL3;HIST1H4K;HIST1H2AK;DDIT4;MAFF;HIST1H4H;HIST1H3H;HIST1H4I
Lanatoside C PC3 *UP*	15/411	1.3002737837675496E−10	1.9027339702465143E−8	10.873997326203208	EGR2;HIST1H2AI;HIST1H4K;HIST1H2AK;CXCR4;ADM;YOD1;NFIL3;MAFF;HIST1H1D;PIM1;HIST1H4H;HIST1H3H;HIST1H4I;HIST1H2BD
Dequalinium chloride HL60 *DOWN*	8/63	2.0044767449747477E−10	2.6398958731317424E−8	38.520242424242426	EGR2;PFKFB3;HILPDA;DDIT4;MAFF;ADM;PLIN2;DHCR7

*COVID-19* Coronavirus 2019, *OA* osteoarthritis.

### ROC curves of hub genes

**Figure 14:**
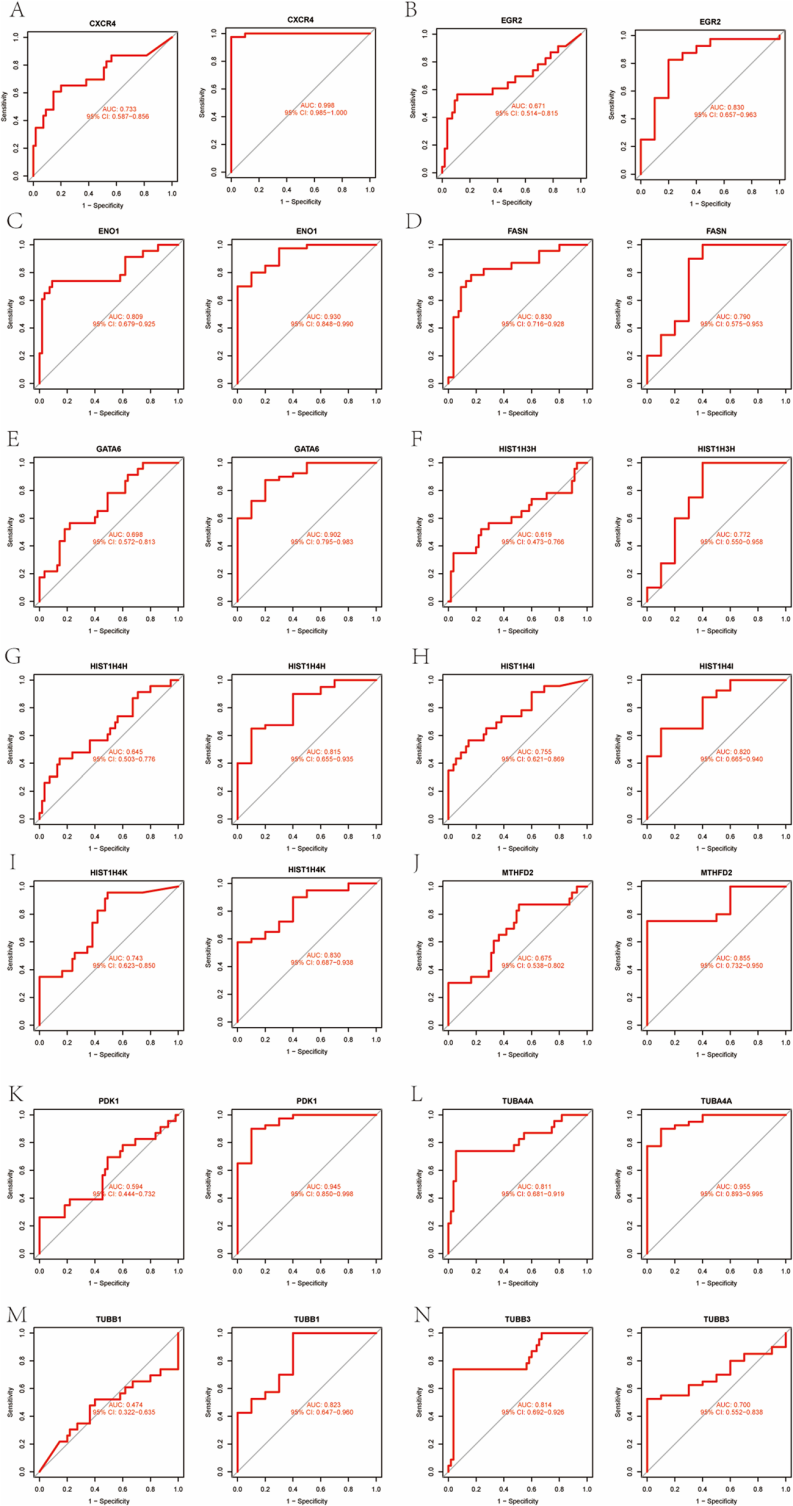
Validation of diagnostic shared hub genes in the GSE147507 and GSE51588. (**A**) CXCR4, (**B**) EGR2, (**C**) ENO1, (**D**) FASN, (**E**) GATA6, (**F**) HIST1H3H, (**G**) HIST1H4H, (**H**) HIST1H4I, (**I**) HIST1H4K, (**J**) MTHFD2, (**K**) PDK1, (**L**) TUBA4A, (**M**) TUBB1, (**N**) TUBB3.

The ROC curve was plotted to evaluate the diagnostic efficacy of 14 key genes (Fig. 14). In the COVID-19 dataset, *CXCR4* (AUC:0.733), *ENO1* (AUC:0.809), *FASN* (AUC:0.830), *HIST1H4I* (AUC:0.755), *HIST1H4K* (AUC:0.743), *TUBA4A* (AUC:0.811) and *TUBB3* (AUC:0.814) exhibited relatively good diagnostic efficiency for distinguishing the patients with COVID-19 from healthy controls. In the OA dataset, CXCR4 (AUC: 0.998); EGR2 (AUC:0.830), ENO1 (AUC:0.930), FASN (AUC:0.790), GATA6 (AUC:0.902), HIST1H3H (AUC:0.772), HIST1H4H (AUC:0.815), HIST1H4I (AUC:0.820), HIST1H4K (AUC:0.830), MTHFD2 (AUC:0.855), PDK1 (AUC:0.945), TUBA4A (AUC:0.955), TUBB1 (AUC:0.823) and TUBB3 (AUC:0.700) displayed preferable diagnostic performance for differentiating OA from healthy individuals. Specifically, in the COVID-19 dataset, *FASN* showed the best diagnostic efficiency for differentiating, while *CXCR4* showed the best differentiating capability in the OA dataset.

## Discussion

There is evidence that patients with osteoarthritis (OA) have a higher prevalence and worse prognosis after COVID-19 infection^11,40^. COVID-19 infection can cause pathological changes in multiple organs, including the musculoskeletal system^18,41^. Some relevant researches suggested that some inflammation and immune response might involve in it^14^. Therefore, we attempted to explore the shared function and pathways between COVID-19 and OA, and to determine the interrelationship between COVID-19 and OA.

In this study, 83 shared DEGs and 14 hub genes (*CXCR4*, *EGR2*, *ENO1*, *FASN*, *GATA6*, *HIST1H3H*, *HIST1H4H*, *HIST1H4I*, *HIST1H4K*, *MTHFD2*, *PDK1*, *TUBA4A*, *TUBB1* and *TUBB3*) have been identified.

Among them, six genes (*CXCR4*, *ENO1*, *FASN*, *GATA6*, *PDK1* and *TUBB3*) have been reported to be associated with the pathological mechanism of COVID-19 and OA. *CXCR4* and *PDK1* involved in the pathogenesis of both COVID-19 and OA. *CXCR4*, shorten for CXC chemokine receptor 4, is a G-protein-coupled receptors (*GPCR*) which can activate a variety of downstream signaling pathways^42^. Scientists has found that *CXCR4* was highly expressed in COVID-19 patients^43^. Reasons are probably that *CXCR4*-positive pre-neutrophils (preNeu) may be released prematurely from bone marrow into blood and infiltrate lung tissue in severe patients^44^. While in OA tissue, *SDF-1/CXCR4* axis has been reported to coordinate the communication between subchondral bone and articular cartilage, which is considered to be a central feature of OA occurrence and development^45^. *PDK1,* Phosphoinositide dependent protein kinase-1, has been found to be associated with apoptosis^46^. In OA, researches demonstrated that *PDK1* could promote apoptosis of chondrocytes via modulating *MAPK* pathway, and has been identified as hub genes of hypermethylation low-expression genes^46,47^. Another investigation revealed that SARS-CoV-2-encoded nucleocapsid protein N could specifically enhance the M-induced apoptosis via interacting with both M and *PDK1*, thereby enhancing M-mediated attenuation of *PDK1-PKB/Akt* interactions^48^. *ENO1* and *TUBB3* were found to be probably related to the progression of OA, while *FASN* and *GATA6* may participate in COVID-19. α-Enolase (*ENO1*) is an enolase isoform widely expressed in almost all mammal cells, characterized as a key glycolytic enzyme and an RNA-binding protein^49^. In inflammatory arthritis, enhanced surface expression of *ENO1* in patient-derived peripheral blood mononuclear cells promotes inflammatory response^50^. It was also found that *ENO1* could promote osteoarthritis progression through interacting with Circular RNA circNFKB1 and sustaining *NF-κB* signaling^49^. *TUBB3* has been confirmed to be the neuron markers induced by the differentiation process in a model which mimicked a pro-nociceptive microenvironment, which could be further investigated in the field of pain in OA. *FASN*, Fatty acid synthase, is a key cellular enzyme in palmitate synthesis^51^. SARS-CoV-2 was expected to increase production of lipid anabolic enzymes including *FASN* by increasing *PI3K/AKT/mTOR/S6K* signaling activity^52^. Scientists also discovered that inhibition of *FASN* and restoration of lipid catabolism could impede replication of coronaviruses closely related to SARS-CoV-2^51^. *GATA6* is a member of a small family of zinc-finger DNA-binding transcription factors, and plays an important role in the regulation of cellular differentiation^53^. Of note, *GATA6* is involved in immune and inflammatory responses by promoting the transcription of *SFTPA* gene. Scientists discovered that both *GATA6* and *SFTPA* genes were upregulated in SARS-CoV-2-infected lungs^54^. Genome-wide CRISPR screens also identified *GATA6* as a pro-viral host factor for SARS-CoV-2 via modulation of *ACE2*^53^. The remaining 8 key genes (*EGR2*, *HIST1H3H*, *HIST1H4H*, *HIST1H4I*, *HIST1H4K*, *MTHFD2*, *TUBA4A*, and *TUBB1*) were less studied in the roles of COVID-19 and OA, emphasizing their importance in future research.

Enrichment analysis in our study indicated that hypoxia-inducible factor (HIF)-1 signaling pathway is a common pathogenesis of COVID-19 and OA. Since the discovery of *HIF-1*, numerous studies on the hypoxia signaling pathway have been performed^55^. The role of *HIF* stabilization during hypoxia has expanded from the induction of a single gene, erythropoietin, to the upregulation of a couple of hundred downstream targets demonstrating the complexity and importance of HIF signaling pathway^55^. Several studies have showed that *HIF-1* signaling pathway could be a potential target for therapeutic interventions of COVID-19^19,52,56^. In an integrated proteo-transcriptomics analysis, *HIF-1* signaling pathway was markedly modulated during the course of the SARS-CoV-2 infection in vitro^52^. Codo et al. discovered that elevated glucose levels favored SARS-CoV-2 infection through a *HIF-1α*/glycolysis-dependent axis^56^. Moreover, inflammatory and reparative activities of lung macrophages are regulated by *β-catenin-HIF-1α* signaling, with implications for the treatment of severe respiratory diseases including COVID-19^19^. *HIF-1* signaling pathway is essential in the homeostasis of multiple tissues in OA as well^57^. For example, Chen et al. found that *HIF-1-VEGF-Notch* mediated angiogenesis in temporomandibular joint osteoarthritis^58^. Another in vitro experiment showed that *HIF‐1α* facilitated osteocyte‐mediated osteoclastogenesis by activating *JAK2/STAT3* pathway^59^. Scientists found that *HIF-1α* maintained mouse articular cartilage stabilization through suppression of *NF-κB* signaling^60^, and the interaction of *HIF1α* and *β-catenin* inhibited matrix metalloproteinase 13 expression so that preventing cartilage damage^61^.

Several chemical agents and drugs have been utilized as potential therapeutic targets against COVID-19 or OA. However, up to now, no drugs were identified to treat individuals with both COVID-19 and OA. In our study, we explored several drugs which could be used as possible targets. Dimethyloxalylglycine is an inhibitor of HIF prolylhydroxylase, which stabilizes and accumulates HIF-1α protein in the nucleus and is an agonist of HIF-1α. Chondrocytes treated with dimethyloxalylglycine could stabilize HIF-1α alleviating osteoarthritis^62^. Cephaeline acted by repressing HIF-1α ± and disturbing intracellular redox homeostasis. Cephaeline were discovered to inhibit SARS-CoV-2 with EC50 values of low nanomolar levels potently^63^. Another extracted drug was Cycloheximide, which is an inhibitor of eukaryotic protein synthesis. Cycloheximide inhibits ferroptosis and inhibits autophagy. In clinical trials, cycloheximide showed potent activity against human coronaviruses^64^.

Although previous studies have respectively explored the pivotal genes associated with each COVID-19 or OA, studies exploring the common molecular mechanisms between the two through bioinformatic approaches are still in vacancy. In this study, for the first time, we explored and identified common DEGs and hub genes of both COVID-19 and OA which may help to further elucidate the pathogenesis of both. However, there are also some limitations in our study. Firstly, the data were downloaded from a public database and input errors could not be assessed. Second, in this study we have used two datasets—one is RNA-seq for COVID-19 and the other one is microarray data for OA. However, microarray data has some limitations over RNA-seq data. Furthermore, the sample sizes, age and sex of COVID-19 and OA are not entirely balanced. Last, this study requires external experimental validation to verify our findings and the function of hub genes needs to be further validated in an in vitro model.

Overall, we identified common DEGs and hub genes for both COVID-19 and OA, and performed multiple bioinformatics analysis based on them. COVID-19 and OA were found to share some common pathogenic mechanism that may be mediated by specific pivotal genes. This study provides a potential horizon for further investigation of the molecular mechanisms, finding novel drugs, developing individual-based therapy for patients with both COVID-19 and OA, and paving the road towards the treatment of long-term COVID-19.

## Data Availability

The datasets presented in this study can be found in GEO (https://www.ncbi.nlm.nih.gov/geo/), an online repository. GSE147507 and GSE51588 datasets were downloaded from the GEO database.

## Abbreviations

OA: Osteoarthritis
COVID-19: Coronavirus disease 2019
SARS-CoV-2: Severe acute respiratory syndrome coronavirus 2
RAS: Renin-angiotensin system
GEO: Gene Expression Omnibus
DEGs: Differentially expressed genes
TFs: Transcription factors
miRNAs: MicroRNAs
GO: Gene ontology
KEGG: Kyoto Encyclopedia of Genes and Genomes
DSigDB: Drug Signatures database
ROC: Receiver operating characteristic
AUC: Area under the curve
CXCR4: CXC chemokine receptor 4
ENO1: α-Enolase
FASN: Fatty acid synthase
AMPK: AMP-activated protein kinase
PDK1: Phosphoinositide dependent protein kinase-1
ECM-GC: Glycated extracellular matrix
DRG: Dorsal root ganglion
HIF: Hypoxia-inducible factor
